# Oviposition Response to Light With Different Spectra in the Crepuscular Moth *Grapholita molesta*

**DOI:** 10.1002/ece3.72096

**Published:** 2025-08-29

**Authors:** Xiaofan Yang, Aihong Ma, Yanran Wan, Yueli Jiang, Hongfan Ran, Yiping Niu, Jiancheng Li

**Affiliations:** ^1^ Institute of Plant Protection, Hebei Academy of Agricultural and Forestry Sciences Key Laboratory of IPM on Crops in Northern Region of North China, Ministry of Agriculture and Rural Affairs of China Baoding Hebei China; ^2^ College of Plant Protection, Hebei Agricultural University Baoding Hebei China; ^3^ Institute of Plant Protection, Henan Academy of Agricultural Sciences Zhengzhou Henan China

**Keywords:** *Grapholita molesta*, light response, oviposition preference, UV, vision

## Abstract

The light spectrum is a critical visual feature influencing insect behavior. The crepuscular moth *Grapholita molesta* (Busck), a significant pest of stone and pome fruits worldwide, has been shown to discriminate variations in brightness/intensity under dim‐light conditions. However, the behavioral responses of *G. molesta* females to various light spectra remain unknown. In this study, we investigated the oviposition preference of *G. molesta* females by assessing eggs laid in light and dark conditions, as well as under two different types of light in two‐choice experiments. Light in the upper canopy of *Prunus persica* is richer in UV light than lower parts characterized by a green light spectrum. When given the choice between light and dark, *G. molesta* females preferred to lay eggs in the illuminated areas over the dark ones. Further experiments revealed that when presented with colored light versus white light of the same intensity, or between two lights of different spectra, *G. molesta* females could discriminate between these light types. They exhibited a strong preference for ultraviolet (UV) light, followed by blue and green light. Notably, this preference for UV light was dependent on intensity, with higher intensity light attracting more female. In cases where spectrum and intensity cues conflicted (e.g., UV light was paired with lights that had 10‐fold or 100‐fold lower intensity), *G. molesta* females still showed a preference for UV light for oviposition. These results indicate that the crepuscular *G. molesta* can differentiate between various light spectra, and the preference for UV light likely aids females in selecting suitable oviposition sites during dusk.

## Graphical abstract

The crepuscular moth *Grapholita molesta* can discriminate differences in light wavelength, exhibiting a strong preference to lay eggs in UV light.

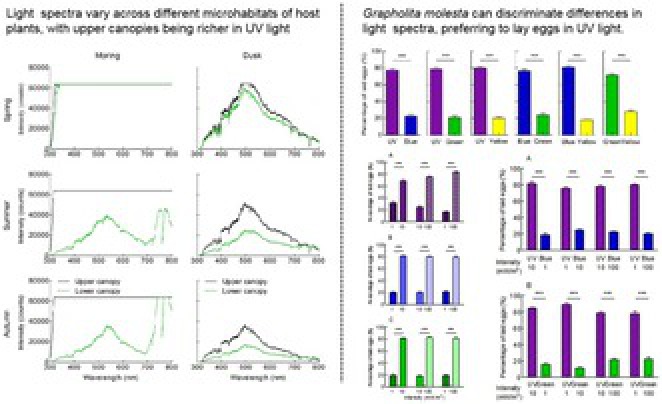

## 1 Introduction

In most insects, gravid females have evolved oviposition behaviors to select appropriate sites within complex environments, thereby maximizing offspring survival, development, and reproductive success (Refsnider and Janzen 2010). Spatial and temporal heterogeneity in environmental cues (including visual, olfactory, and tactile features) drives these decisions (Kelber 1999; Dweck et al. 2013; Karageorgi et al. 2017; Li et al. 2020). Among these, light spectral composition is a critical visual feature influencing habitat assessment for oviposition (Théry 2001). For instance, *Cydia strobilella* females show a preference for long‐wavelength light during egg‐laying (Jakobsson et al. 2017). *Drosophila* exhibit strong phototaxis toward short wavelengths, particularly ultraviolet (UV) rather than visible light (Gao et al. 2008; Yamaguchi et al. 2010; Karuppudurai et al. 2014; Guntur et al. 2015; Lazopulo et al. 2019; Currier et al. 2023), but switch away from UV light during oviposition (Zhu et al. 2014), which probably reflects different ecological needs. An increased understanding of spectral preferences in oviposition behavior could inform the development of species‐specific light‐based attractants or repellents for pest management strategies.

The crepuscular oriental fruit moth, *Grapholita molesta* (Busck) (Lepidoptera: Tortricidae), is a significant pest of stone and pome fruits worldwide, causing substantial economic damage to peach ( *Prunus persica* L.), pear ( *Pyrus communis* L.), plums ( *Prunus salicina* Lindl.) and apple ( *Malus domestica* Borkh.) (Ammagarahalli and Gemeno 2015). Insect control methods based on the sensory physiology mediating oviposition are very appealing as chemical pesticides are non‐specific and environmentally harmful. Gravid females prefer to lay eggs on the young leaves at the top of host plants around dusk (Myers et al. 2006; Yang et al. 2022). While olfactory cues, specifically host volatiles, have been extensively documented as long‐range orientation signals for distinguishing between host and non‐host plants or host species (Najar‐Rodriguez et al. 2013; Piñero and Dorn 2009; Natale et al. 2003, 2004; Il'Ichev and Kugimiya 2009), visual cues become more important for perception in microhabitats. The light characteristics, such as spectrum, can vary in different microhabitats of host plants. For example, the top of plants receiving direct sunlight is likely richer in UV than the lower parts, which are characterized by a “yellow‐green” light spectrum (Endler 1993; Théry 2001). Could the light spectrum affect the selection of oviposition sites for *G. molesta*?

Our recent studies demonstrate that *G. molesta* females can visually discriminate between plants in microhabitats to guide oviposition even in dim light, exhibiting a preference for higher brightness/intensity (Yang et al. 2020, 2022). In Yang et al. (2020), we also tested the response to six different colors, finding a preference for green and orange over yellow, red, blue, and purple. Sun et al. (2014) reported that *G. molesta* is more responsive to green and violet light. While these studies establish the color preferences within the visible spectrum, the behavioral responses of *G. molesta* to UV light remain uncharacterized. Electroretinogram (ERG) studies additionally suggest that three photoreceptor classes are likely present in the compound eyes of *G. molesta*, with sensitivity peaks at UV, blue, and green wavelengths (Martín‐Gabarrella et al. 2023). We hypothesize that *G. molesta* females exhibit strongly positive or negative phototaxis toward light of specific spectrum, such as UV, to facilitate recognition of oviposition sites.

In this study, we characterized the behavioral responses of *G. molesta* females to different light spectra. To avoid the potential interaction of color and intensity caused by colored papers (Yang et al. 2020), we selected the colored lights in this experiment, as their intensity can be precisely controlled. We examined the oviposition preferences of *G. molesta* females in two‐choice experiments, comparing light versus dark conditions, as well as colored versus white light of the same intensity. Additionally, we evaluated the attraction to pairs of light sources that differed in spectrum, intensity, or both, to determine whether specific‐spectrum light plays an important role in guiding oviposition site selection in this crepuscular species.

## 2 Materials and Methods

### 2.1 Insect Rearing

A colony of *G. molesta* was established from wild‐caught larvae collected in a peach orchard in Shunping, Hebei, China, in August 2020. The colony was maintained in the artificial climate chambers at 26°C ± 1°C, 70% ± 5% relative humidity (RH) under a 15 h:9 h light: dark cycle with a light intensity of 6000 lx during photophase. Larvae were initially mass‐reared on apples for the first three instars and then transferred to an artificial diet (Yang et al. 2016), and adults were fed a 10% honey solution on cotton balls. Newly emerged adults were sexed and kept individually in glass tubes (1.8 cm diameter, 8 cm height) without access to food or water. Groups of ten 2‐day‐old females and males were paired 3–4 h before the onset of the scotophase. During the ensuing hours, copulating pairs were removed gently and separated 12 h later. Mated 3‐day‐old females were collected for oviposition preference experiments.

### 2.2 Light Measurement Within Canopy

Spectral measurements were conducted in a peach orchard (cv. Green No. 9) of 1.4 ha in Shunping County of Hebei during the spring (April 20), summer (June 28) and autumn (August 26) of 2021. These days corresponded to the peak oviposition periods of overwintering, second‐generation, and fourth‐generation *G. molesta* adults, respectively. The orchard consisted of 14‐year‐old trees spaced 6 × 5 m apart, with an average height of 3.2 m and a canopy diameter ranging from 2.8 to 3.1 m. The trees were trained in an open‐center shape with three main branches. Ambient light spectrum was measured at both the upper and lower parts of the peach canopy using a spectrometer (USB 4000, Ocean Optics Inc., Dunedin, FL, USA) and OceanView 2.0 software. Measurements were taken in the morning (10:00) and around dusk (0.5 h before sunset). The upper canopy, exposed to direct sunlight, represented open conditions, while the lower canopy was largely shaded. Five randomly selected sites within the canopy were chosen.

### 2.3 Two‐Choice Oviposition Experiments

**FIGURE 1:**
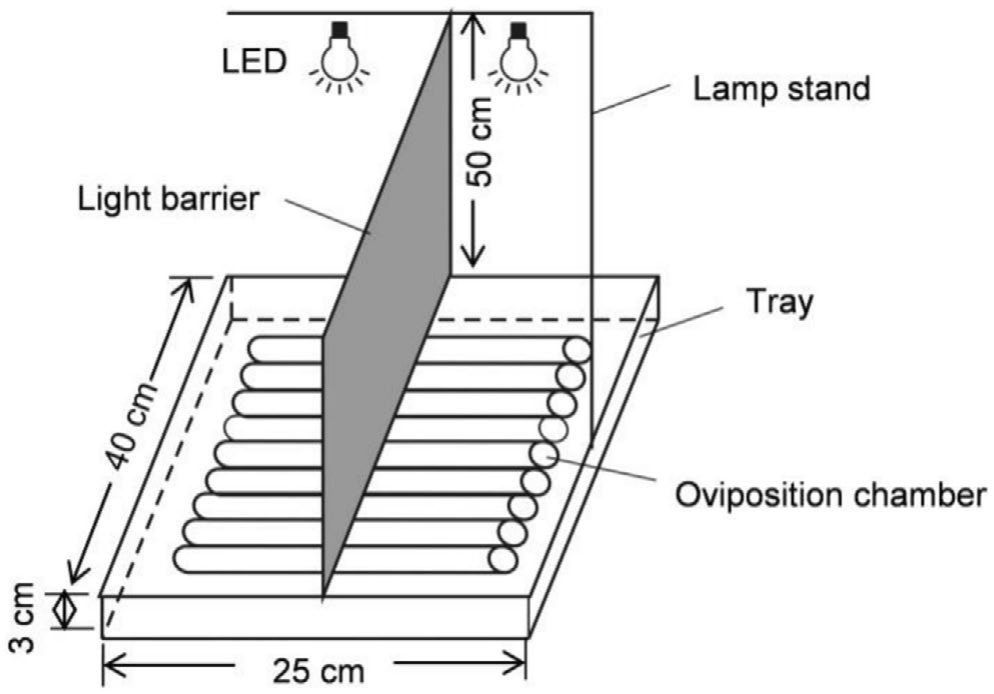
Experimental setup. The oviposition chamber, designed to test the oviposition preference of *Grapholita molesta*, consisted of 20 glass tubes. An LED light source was positioned 50 cm above each half of the chamber to provide light stimuli, effectively dividing the chamber into two oviposition substrates with different light environments. Moths were allowed to move freely within the chamber to choose an oviposition site.

**FIGURE 2:**
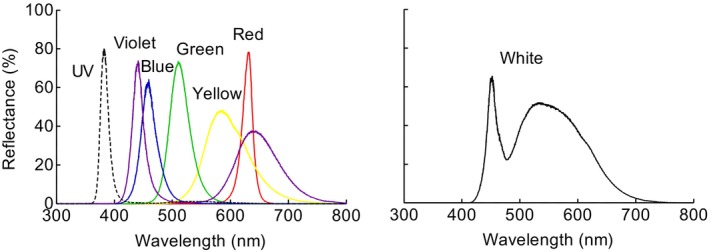
Emission spectra of the illuminating LEDs used in this study.

All experiments were conducted in an oviposition chamber (Figure 1) in a dark room at 26°C ± 1°C, 60% ± 5% RH. The oviposition chamber, placed on a white plastic tray (40 × 25 × 3 cm), consisted of 20 horizontal glass tubes (2 cm diameter, 14 cm height), allowing up to 20 moths to be housed simultaneously. The smooth surface of the glass tubes provided a basic thigmotactic stimulus to encourage oviposition. Two ends of the glass tubes were fitted with gauzes after the moth was introduced to the tube, preventing moths from escaping and maintaining sufficient air humidity throughout the tubes to elicit oviposition. A black square plate (40 cm long, 50 cm height) was positioned in the center of the oviposition chamber to act as a light barrier to avoid light transmission. During the experiments, the chamber was illuminated from 50 cm above by two custom LED lamps (18 W, 14 cm diameter; Guangzhou Xinyuan Optoelectronic Technology Co. LTD, Guangzhou, China), which differed in spectrum, intensity, or both. This setup allowed the two halves of oviposition chamber to be exposed to different light stimuli. In some cases, only one LED was activated illuminating one half of the chamber while leaving the other half in darkness. The LEDs used in this study were the following: UV (*λ*_max_ = 380 nm), violet (*λ*_max_ = 430 nm), blue (*λ*_max_ = 455 nm), green (*λ*_max_ = 510 nm), yellow (*λ*_max_ = 580 nm), red (*λ*_max_ = 630 nm) and white (broad‐spectrum with two emission peaks at blue and green‐yellow). Spectrum measurements were taken with a spectrometer (USB 4000, Ocean Optics Inc., Dunedin, FL, USA) using OceanView 2.0 software (Figure 2). Light intensity was measured at the half center of the chambers using a radiometer (IL1700, International Light Research, Peabody, MA, USA) and adjusted by neutral density filters (Melles Griot, Rochester, NY, USA) and slightly lowering height of the lamps.

Twenty 3‐day‐old mated females were introduced into the oviposition chamber and allowed to lay eggs individually in glass tubes for 15 h overnight, in the absence of host volatiles or sugar solution. The following morning, the females were removed, and the number of eggs laid on both halves of each tube was recorded. All experiments began at 17:00, 3 h before the onset of the scotophase (when the moths start to oviposit). When presenting light stimuli to moths, the chamber was thoroughly cleaned with 75% alcohol. To minimize positional bias, the positions of the LEDs were swapped in a pseudo‐randomed order during the oviposition preference experiments. Each experiment was repeated eight times.

### 2.4 Experiment 1: Fast Phototaxis

To test the oviposition response of *G. molesta* to light, females were first exposed to a choice between light and dark conditions, called “fast phototaxis” (Yamaguchi et al. 2010). In this way, only one half of the oviposition chamber was illuminated by an LED positioned above. Seven different light spectra including UV, violet, blue, green, yellow, red and white light were tested. These spectra were selected because they cover a broad range of wavelengths from UV to visible light, encompassing the natural lighting conditions encountered by *G. molesta* in its living environment. The light intensity for all LEDs was set to 1 mW/m^2^, corresponding to late twilight.

### 2.5 Experiment 2: Response to Light Spectrum

To examine whether and how light spectrum influences the oviposition behavior of *G. molesta*, we conducted two experiments to test the preferences between two lights of different spectra in same intensity (Zhu et al. 2014). In the first experiment, individual females housed in glass tubes were exposed to a choice between colored light (UV, violet, blue, green, yellow or red) and white light. In the second experiment, we focused on the preferred light spectra identified in the first experiment (UV, blue, green, and yellow) and tested pairwise comparisons between these spectra: UV versus blue, UV versus green, UV versus yellow, blue versus green, blue versus yellow, and green versus yellow. Both colored and white light were set to 1 mW/m^2^.

### 2.6 Experiment 3: Response to Light Intensity

To further characterize the light response of *G. molesta*, we tested the oviposition preference between two lights differing only in intensity by factors of 10 or 100. Three light intensities (100, 10, and 1 mW/m^2^, ranging from early to late twilight) were tested based on the natural light conditions experienced by wild *G. molesta* during the oviposition process. Specifically, females were given a choice between dimmer and brighter light: 1 versus 10 mW/m^2^, 10 versus 100 mW/m^2^, and 1 versus 100 mW/m^2^. The tested light spectra included UV, blue and green light, which were chosen based on the results from experiments of “light response to spectrum”.

### 2.7 Experiment 4: Response to Light Spectrum and Intensity

To test the relative contributions of spectrum and intensity to the light response of *G. molesta*, we compared oviposition preferences between two lights differing in both spectrum and intensity. Females were exposed to choices between UV and blue or UV and green at different intensities, ranging from 1, 10 to 100 mW/m^2^.

### 2.8 Data Analysis

Chi‐squared tests were used to assess the differences between eggs laid in light and dark, or in two different light in the two‐choice experiments. Females that has not laid eggs were recorded but excluded from analysis. All statistical analyses were conducted using SPSS 19.0 (SPSS Inc., Chicago, IL, USA).

## 3 Results

### 3.1 Light Measurement Within Canopy

**FIGURE 3:**
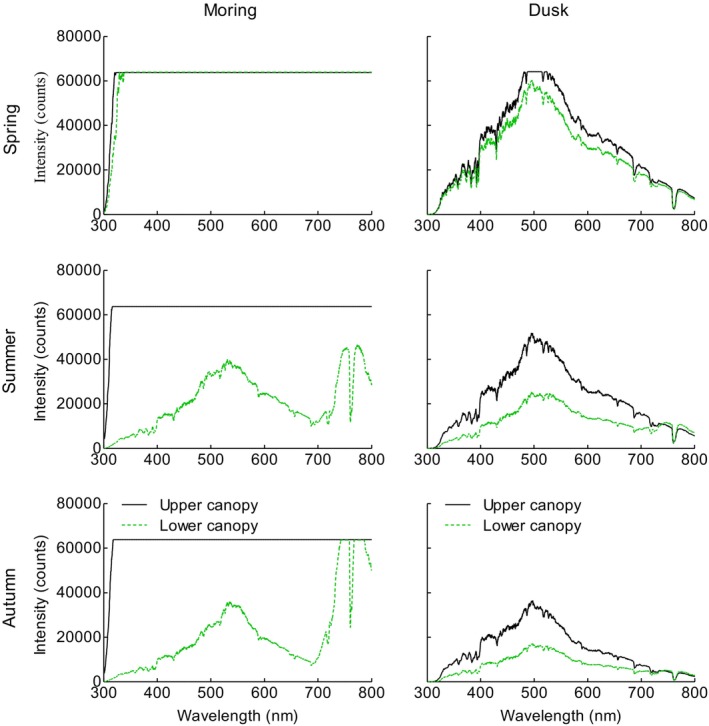
Ambient light spectrum at upper and lower canopy of peach tree in spring, summer, and autumn.

In spring, the ambient light spectra in upper and lower canopies were similar, exhibiting a rapid increase above 300 nm, followed by a rough flat between 320 and 800 nm in morning. Around dusk, the spectra showed a strong reduction at short and long wavelengths, with a broad peak between 480 and 540 nm (Figure 3A). No significant differences were observed between summer and autumn (Figure 3B,C). In summer and autumn mornings, the ambient light spectra in the lower canopy was enriched in middle wavelengths, ranging from 490 to 560 nm, which differed from that of in upper canopy. Around dusk, however, the spectra in both upper and lower canopies showed a similar peak at 500 nm. These findings indicate that light characteristics in different canopies were different, with the upper canopy being richer in UV light compared to the lower parts, which is characterized by a green light spectrum, potentially exhibiting a strong effect on oviposition site selection of *G. molesta*.

### 3.2 Fast Phototaxis

**FIGURE 4:**
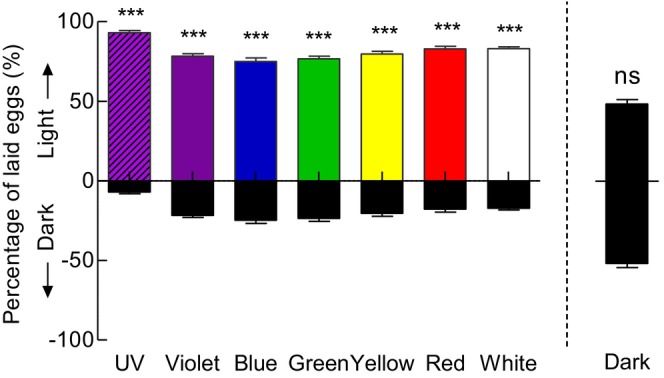
Oviposition preference of *Grapholita molesta* in light versus dark choices. Data shown as mean ± SEM; *n* = 8 experiments, 160 moths. ***A significant difference at *p* < 0.001; ns indicates no significant difference at *p* > 0.05 (chi‐squared test).

When presented with a choice between light versus dark conditions, *G. molesta* females strongly preferred to lay eggs in the illuminated halves over the dark halves (Figure 4). Oviposition selection frequencies for UV, violet, blue, green, yellow, red, and white light were 93.13%, 78.49%, 75.09%, 76.70%, 79.65%, 82.75%, and 82.92%, respectively (*p* < 0.001).

### 3.3 Response to Light Spectrum

**FIGURE 5:**
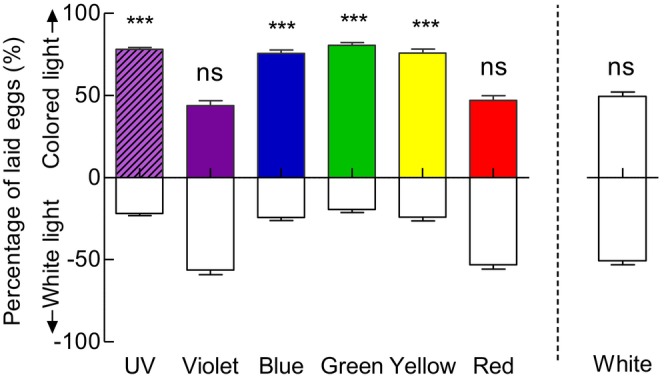
Oviposition preference of *G. molesta* in choices between colored light and white light of the same intensity (1 mW/m^2^). Data shown as mean ± SEM; *n* = 8 experiments, 160 moths. ***A significant difference at *p* < 0.001; ns indicates no significant difference at *p* > 0.05 (chi‐squared test).

When given a choice between colored and white light of the same intensity, *G. molesta* females showed significant preferences for UV, blue, green, and yellow light compared with white light, with oviposition frequency > 75% (*p* < 0.001). In contrast, attraction to violet or red light was not significantly different from that to white light (Figure 5) (*p*_violet_ = 0.09; *p*_red_ = 0.258). Under complete white light, eggs were evenly distributed throughout the chamber (*p* = 0.77).

**FIGURE 6:**
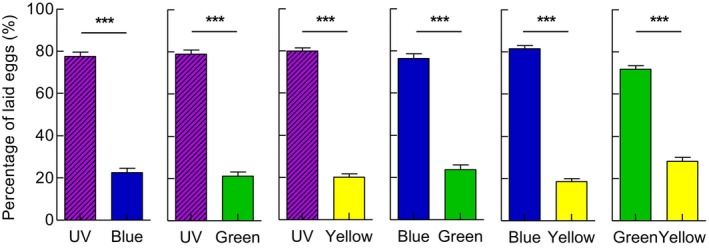
Oviposition preference of *G. molesta* in choices between two lights differed in spectrum but in the same intensity (1 mW/m^2^). Data shown as mean ± SEM; *n* = 8 experiments, 160 moths. ***A significant difference at *p* < 0.001 (chi‐squared test).

When given the choice between two lights of different spectra, females significantly preferred UV light, with a more than 77% oviposition frequency (*p* < 0.001), followed by blue and green light (Figure 6) (*p* < 0.001).

### 3.4 Response to Light Intensity

**FIGURE 7:**
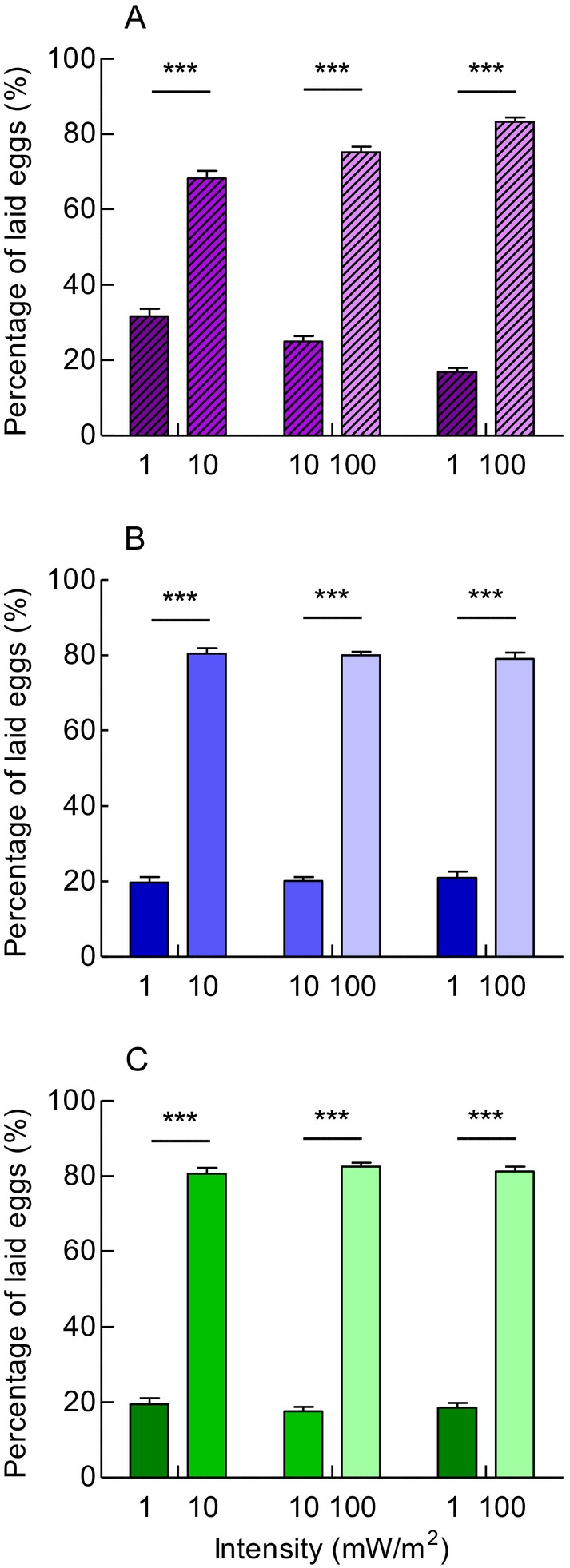
Oviposition preference of *G. molesta* in choices between two lights of different intensities. (A) Preference for different UV light intensities. (B) Preference for different blue light intensities. (C) Preference for different green light intensities. Data shown as mean ± SEM; *n* = 8 experments, 160 moths. ***A significant difference at *p* < 0.001 (chi‐squared test).

In all choices between two UV lights differing in intensity by a factor of 10 or 100, *G. molesta* females showed a strong preference to lay eggs in the higher‐intensity light than in lower‐intensity light (Figure 7A) (*p* < 0.001). Similarly, females significantly preferred higher‐intensity blue and green light over their lower‐intensity counterparts (Figure 7B,C) (*p* < 0.001). These results suggest that the light preference of *G. molesta* is intensity‐dependent, with higher‐intensity light eliciting stronger attraction.

### 3.5 Response to Light Spectrum and Intensity

**FIGURE 8:**
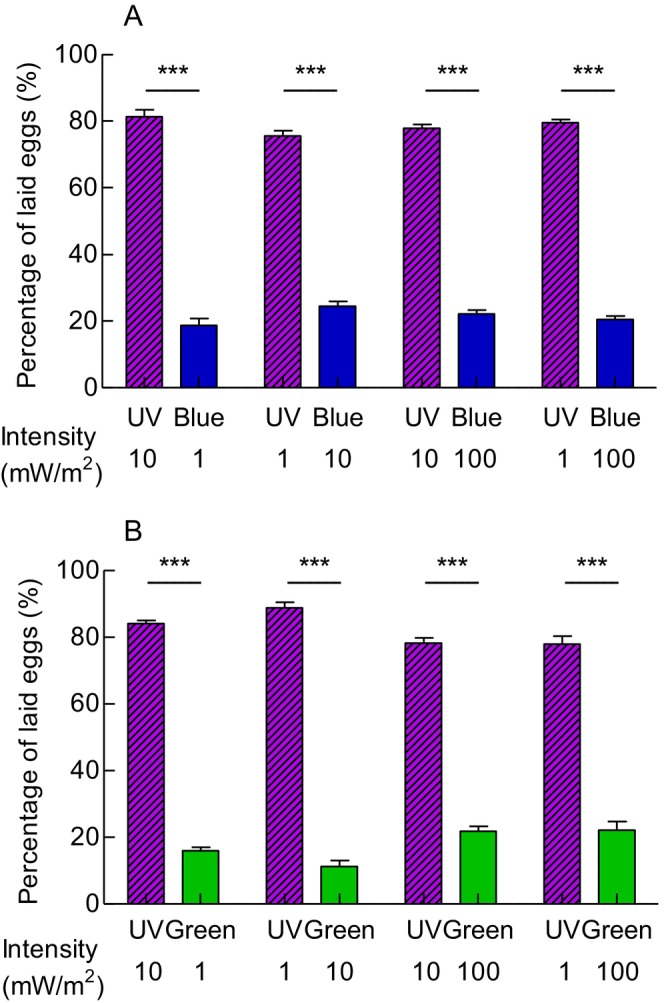
Oviposition preference of *G. molesta* in choices between two lights differed in both spectrum and intensity. (A) Preference between UV and blue light. (B) Preference between UV and green light. Data shown as mean ± SEM; *n* = 8 experments, 160 moths. ***A significant difference at *p* < 0.001 (chi‐squared test).

To compare the relative contributions of spectrum and intensity, *G. molesta* females were exposed to choices between two lights differing in both spectrum and intensity. In UV versus blue choices, females consistently showed a strong preference for UV light over blue light, even when the UV light was paired with a 10‐fold or 100‐fold lower intensity (Figure 8A, *p* < 0.001). Similarly, females preferred UV light over green light, regardless of intensity differences (Figure 8B, *p* < 0.001). Taken together, these findings indicate that *G. molesta* females are more strongly attracted to UV light than to light intensity.

## 4 Discussion

This work was a continuation of our previous work, which demonstrated that *G. molesta* can discriminate differences in brightness/intensity under dim light, and that the preference for higher brightness/intensity may help females to choose oviposition sites (Yang et al. 2020, 2022). In addition to intensity, spectral composition is another fundamental characteristic of light. However, our earlier study was unable to conclusively establish spectrum discrimination in *G. molesta*, because the colored papers (red, orange, yellow, green, blue and purple) used varied in intensity and did not include UV light, which is known to guide many important behaviors in insects (Yang et al. 2020). To address this limitation, we conducted two‐choice experiments using colored lights. When the intensity of two lights was the same, *G. molesta* showed a different response to the light of different spectra under dim light corresponding to late twilight, exhibiting a strong preference for UV light, followed by blue and green light (Figures 4 and 5). This was not quite consistent with our previous results, where green and orange are more attractive than purple, blue, yellow, and red (Yang et al. 2020). These results indicated that UV, blue, and green sensitive photoreceptors appear to be contained in the compound eyes of *G. molesta*.

When the spectrum of two lights was the same, the oviposition responses of *G. molesta* to light (e.g., UV, blue and green) were intensity‐dependent, with higher‐intensity light eliciting stronger attraction (Figure 6). This aligns with our previous findings using white light (Yang et al. 2022). However, when spectrum and intensity cues were presented in conflict, such as UV light being paired with 10‐fold or 100‐fold lower intensity, *G. molesta* females were still preferentially attracted to UV light, regardless of intensity (Figure 7). This preference for UV light may help the crepuscular moth find optimal oviposition sites around dusk. To discriminate spectral differences between microhabitats on host plants, *G. molesta* likely relies on a highly sensitive visual system. Indeed, our recent work has confirmed that *G. molesta* possesses superposition compound eyes, which enhance visual sensitivity and enable efficient function in dim light (Yang et al. 2024).

Why do *G. molesta* females exhibit a UV attraction for oviposition? One possible explanation is that the upper canopy of host plants, where *G. molesta* females preferentially lay eggs, is typically richer in UV light (Figure 3). The preference of UV over visible light may thus help females discriminate the upper canopy from lower parts to oviposit. Presumably, richer UV light is likely related to the plant quality, helping to optimize the offspring development and survival of *G. molesta*. Previous studies have shown that host plants exposed to UV light often contain richer nutritional resources, such as higher nitrogen content, resulting in faster development, increased survival rates, and greater fecundity in insects (Huberty and Denno 2006; Osier and Jennings 2007; Uyi et al. 2015). Alternatively, *G. molesta* females may exhibit innate UV phototaxis, leading to indiscriminate egg‐laying near UV sources. Strong UV attraction is common in crepuscular and nocturnal moths, including *Plodia interpunctella*, *Helicoverpa armigera*, *Spodoptera exigua* , *Spodoptera frugiperda* (Cowan and Gries 2009; Meng et al. 2010; Somers‐Yeates et al. 2013; van Grunsven et al. 2014; Liu et al. 2018; Kim et al. 2019; Wang et al. 2022; Yao et al. 2023). However, Sun et al. (2014) reported sex‐specific light responses in *G. molesta*, with females showing stronger attraction. Future studies should compare UV responses between sexes and mating states (virgins and mated females) to examine whether the UV serves as an oviposition cue or a general attractant. Beyond light properties, oviposition behavior is influenced by genetic factors, hormonal regulation (e.g., juvenile hormone), and reproductive nutrition (e.g., vitellogenin) (Truman and Riddiford 2007; Bai et al. 2024). These intrinsic factors may interact with external light cues to modulate oviposition decisions.

Light response to spectrum can vary with circadian rhythm. For instance, *Drosophila* prefer green light in the early morning and late afternoon but reduce this preference at midday (Lazopulo et al. 2019). Similarly, diurnal *Aedes aegypti* are attracted to UV light during the day, while nocturnal *Anopheles coluzzii* exhibit behavioral avoidance of UV light during the day and change to attraction during the night (Baik et al. 2020; Au, Foden, et al. 2022; Au, Liu, et al. 2022). Given that the behavior of *G. molesta* is subject to circadian regulation, further studies should investigate the time‐of‐day variance in light response to fully understand its spectral preference.

In conclusion, our results suggest that the crepuscular moth *G. molesta* females can discriminate between light spectra and intensity, exhibiting a strong preference for UV light and higher intensity. This light response likely helps the crepuscular moth to lay eggs on the upper canopy of host plants during dusk. Due to the ineffectiveness of conventional insecticides against *G. molesta* larvae in cryptic habitats (Kanga et al. 2003), understanding oviposition response to different light spectra could inform the development of alternative monitoring and management strategies, such as UV traps, to reduce infestation levels in orchards (Kim et al. 2019). Additionally, behavioral response to UV light is mediated both by UV‐sensitive opsins and cryptochrome (CRY) (Gao et al. 2008; Guntur et al. 2015; Baik et al. 2017, 2019). Further research should investigate the relative contributions of these phototransduction systems in *G. molesta* to elucidate the molecular mechanism underlying UV attraction.

## Author Contributions

**Xiaofan Yang:** conceptualization (lead), data curation (lead), formal analysis (lead), investigation (lead), methodology (equal), project administration (lead), visualization (lead), writing – original draft (lead), writing – review and editing (equal). **Aihong Ma:** data curation (supporting), formal analysis (supporting), investigation (supporting), methodology (equal), writing – review and editing (equal). **Yanran Wan:** conceptualization (supporting), data curation (supporting), investigation (supporting), methodology (equal), supervision (equal), visualization (supporting), writing – review and editing (equal). **Yueli Jiang:** data curation (supporting), formal analysis (supporting), methodology (equal), software (equal), supervision (equal), visualization (supporting), writing – review and editing (equal). **Hongfan Ran:** data curation (supporting), methodology (equal), software (equal), writing – review and editing (equal). **Yiping Niu:** formal analysis (equal), methodology (equal), software (equal), supervision (equal), writing – review and editing (equal). **Jiancheng Li:** conceptualization (equal), methodology (equal), project administration (supporting), resources (equal), supervision (equal), visualization (supporting), writing – review and editing (equal).

## Conflicts of Interest

The authors declare no conflicts of interest.

## Supporting information


**Data S1:** ece372096‐sup‐0001‐Supinfo1.rar.

## Data Availability

The data that support the findings of this study are available in the Supporting Information of this article.
